# CXCL10 blockade protects mice from cyclophosphamide-induced cystitis

**DOI:** 10.1186/1476-8518-6-6

**Published:** 2008-10-28

**Authors:** Senthilkumar K Sakthivel, Udai P Singh, Shailesh Singh, Dennis D Taub, Kristian R Novakovic, James W Lillard

**Affiliations:** 1 Department of Pathology, Emory University School of Medicine, Atlanta, GA, USA; 2 Deparment of Pathology, Microbiology and Immunology, University of South Carolina School of Medicine, Columbia, SC 29208, USA; 3 Department of Microbiology and Immunology, University of Louisville, Louisville, KY, USA; 4 Laboratory of Immunology, Gerontology Research Center, National Institute on Aging, Baltimore, MD, USA; 5 Department of Urology, University of Louisville, Louisville, KY, USA; 6 Department of Microbiology, Biochemistry, and Immunology, Morehouse School of Medicine, Atlanta, GA, USA

## Abstract

**Background:**

Alterations in serum CXCR3 ligand levels were examined in interstitial cystitis (IC) patients; similar expression patterns in serum as well as CXCR3, CXCR3 ligands, and cytokines expressed by peripheral and local leukocyte subpopulations were characterized during cyclophosphamide (CYP)-induced acute cystitis in mice.

**Results:**

Serum levels of monokine-induced by interferon-γ (IFN-γ) (MIG/CXCL9), IFN-γ-inducible protein-10 (IP-10/CXCL10), and IFN-γ-inducible T cell α chemoattractant (I-TAC/CXCL11) were elevated in patients with IC. These clinical features closely correlated with CYP-induced cystitis in mice. Serum levels of these CXCR3 ligands and local T helper type 1 (Th1) cytokines were also increased. We demonstrate that CXCR3 as well as CXCL9, CXCL10 and CXCL11 mRNA were significantly expressed by urinary bladder lymphocytes, while CXCR3 and CXCL9 transcripts were significantly expressed by iliac lymph node leukocytes following CYP treatment. We also show that the number of CD4^+^ T cells, mast cells, natural killer (NK) cells, and NKT cells were increased at systemic (spleen) and mucosal (urinary bladder and iliac lymph nodes) sites, following CYP-induced cystitis in mice. Importantly, CXCL10 blockade attenuated these increases caused by CYP.

**Conclusion:**

Antibody (Ab)-mediated inhibition of the most abundant serum CXCR3 ligand, CXCL10, in mice decreased the local production of CXCR3 ligands as well as Th1 cytokines expressed by local leukocytes, and lowered corresponding serum levels to reduce the severity of CYP-induced cystitis. The present study is among the first to demonstrate some of the cellular and molecular mechanisms of chemokines in cystitis and may represent new drug target for this disease.

## Background

IC is a complex disease with symptoms including chronic urinary bladder inflammation, characterized by increased urinary frequency, urgency as well as suprapubic, bladder, and pelvic pain. Estimates indicate there are ~1 million IC cases in the United States annually, with 90% of sufferers being women [1-3]. While the precise etiology of IC is not known, a number of causes have been suggested, which include: infection, autoimmunity, urinary toxins, urinary bladder wall epithelial permeability and neurogenic inflammation [4,5]. In several reports, CYP-induced cystitis has been used as a model for IC, because these two conditions share some unique aspects. Both IC and CYP-induced cystitis are non-infectious, cause urinary bladder inflammation, and disrupt the urothelium as well as electrochemical, neurochemical, and micturition reflex elements [6-11]. Indeed, cystitis produced by the administration of CYP is a result of renal excretion of hepatic metabolites, particularly acrolein that contributes to hemorrhagic cystitis and induction of prostaglandins via cyclooxygenase-2 and nitric oxide for the stimulation of cholinergic and inflammatory responses [12-15].

Chemokines have emerged as major factors in inflammatory diseases. These chemotactic cytokines are also considered neuromodulatory agents [16,17]. Further characterization is necessary to better delineate the molecular and cellular inflammatory mechanisms of CYP-induced cystitis. Recently, CX3CR1 and CX3CL1 expression by cells of the urinary bladder have been shown to be enhanced following CYP-induced cystitis [18]. Other chemokines may also be involved in the pathology of cystitis. For example, CXCL9, CXCL10, and CXCL11 bind to a common receptor, CXCR3. These chemokines stimulate and attract CXCR3^+ ^monocytes/macrophages, T cells, NK cells, mast cells and dendritic cells. Many of these leukocytes have been suggested to have a role in cystitis. To this end, CXCR3 expression and CXCL10-signaling by sensory neurons correlate with the maintenance phase of persistent pain [19]. CXCL10 expression has also been shown to be elevated in bladders following cancer therapy and febrile urinary infection [20,21].

While there are no mouse models that precisely replicate either the classical or non-ulcerative forms of IC, the CYP-induced cystitis model used in this study shares many features of the human (non-ulcerative) disease including: urinary bladder inflammation, mastocyte infiltration, and urothelium disruption. This study correlates IC patient serum CXCR3 ligand levels with those of disease free donors as well as with serum from mice with CYP-induced cystitis. Serum CXCL9, CXCL10, and CXCL11 levels of CYP-treated mice given control Ab were compared to similar mice that received anti-CXCL10 Ab. Urinary bladder histology as well as mucosal (iliac lymph node and urinary bladder) and peripheral (spleen) leukocyte expression of CXCR3, CXCL9, CXCL10, and CXCL11 mRNA transcripts as well as CD4^+ ^T cell, mast cell, and NK/NKT cell numbers were compared among naïve and CYP-treated mice given control or anti-CXCL10 Abs. We show that serum levels of CXCL9, CXCL10, and CXCL11 were increased in IC patients. These clinical trends were consistent with CYP-induced cystitis in mice; CXCR3 ligands were elevated in mouse serum as well as urinary bladder and iliac lymph node lymphocytes. Importantly, cystitis severity was reduced when anti-CXCL10 Ab was administered during the development of CYP-induced (acute) cystitis in mice. This study is among the first to suggest that modulation of chemokine interactions can affect the outcome of CYP-induced cystitis.

## Methods

### Patients

A total of 48 age-matched serum samples were collected by Clinomics Biosciences, Inc. (now Cytomyx Holdings, PLC) from a cohort of 32 untreated female patients with chronic and relapsing IC with a mean age of 41.6 years ranging from 31 to 76. In addition, 16 serum samples from unaffected, healthy female donors with a mean age of 47 years ranging from 38 to 62 were collected. Healthy donors had no active urologic disease or symptoms at the time of blood collection. The diagnosis of IC was based strictly on the criteria set forth by the National Institute of Diabetes, Digestive, and Kidney Disease [22]. No subject had a urinary tract infection, hydro-distension, or any intravescical treatment for at least 12 weeks prior to blood collection. All subjects gave written informed consent and were approved by the Clinomics Biosciences Research Ethics Committee (Pittsfield, MA, USA). Subsequently, the University of Louisville Institutional Review Board (IRB) approved the use of these diagnostic specimens in accordance with the Department of Health and Human Service Policy for the Protection of Human Research Subjects 45 CFR 46.101(b) 2 and use of archived de-identified materials.

### Animals

Female BALB/c mice aged 8 to 12 weeks were purchased from Jackson Laboratories (Bar Harbor, ME). Female New Zealand rabbits aged 4 to 5 months (Myrtle's Rabbitory; Thompson Station, TN) were used to generate anti-CXCL10 Abs. Animals were housed and maintained under specific pathogen-free housing. Experimental groups consisted of five mice and each study was repeated three times.

### Anti-CXCL10 Ab generation and treatment

Recombinant mouse CXCL10 was purchased from PeproTech (Rocky Hill, NJ). The endotoxin level of this chemokine was quantified to be < 5 EU/mg by the chromogenic *Limulus *amebocyte lysate assay (Cape Cod, Inc. Falmouth, MS). CXCL10 plus complete Freund's or incomplete Freund's adjuvants (Sigma, St. Louis, MO) were used to generate anti-CXCL10 Ab titers of ~1:2 × 10^6^. Pre-immune or anti-CXCL10 Ab was purified using an IgG isotype-specific protein A column (Pierce Biotechnology, Rockford, IL). The specificity of this IgG Ab was determined by direct enzyme-linked immunosorbent assay (ELISA). No cross-reactivity was detected when tested against other chemokines (CXCL8, CXCL9, CXCL11, CXCL12, CXCL13, CCL2, CCL3, CCL4, CCL5, CCL7, CCL8, CCL11, and XCL1) or cytokines (IL-2, IL-4, IL-5, IL-6, IL-10, IL-12, and TNF-α) (PeproTech, Rocky Hill, NJ). Anti-CXCL10 Ab titers (or control Ab) were adjusted to 1:4 × 10^5 ^(i.e., 50 times dilution) in phosphate-buffered saline (PBS) and subsequently used as "Ab solutions" for treatment.

### CYP-induced cystitis and Ab treatment

CYP administered every 3 days for 10 days can lead to chronic cystitis [23]. In this study, a single intraperitoneal injection of CYP was administered to elicit acute cystitis within 5 days [18]. CYP was obtained from ICN Biomedicals, Inc. (Aurora, OH). Mice of positive control Ab- and anti-CXCL10 Ab-treated groups received intraperitoneal injections of CYP (300 mg/kg) in 200 μl of PBS to induce cystitis. Acute cystitis was studied during the initial 5 days following CYP treatment. 24 hrs before treatment with CYP and every 48 hrs thereafter, mice received 200 μl intraperitoneal injections of either anti-CXCL10 or control Ab solutions. The negative control groups (without CYP treatment) also received anti-CXCL10 or control Ab solutions.

### CXCL9, CXCL10 and CXCL11 ELISAs

Serum concentrations of human and mouse CXCL9, CXCL10, and CXCL11 were determined by ELISA (R&D Systems, Minneapolis, MN), according to the manufacturer's instructions. Each sample was analyzed in triplicate and compared with a standard curve using ELISA techniques to measure CXCL9, CXCL10, and CXCL11 concentrations. In brief, samples and standards were added to the wells and incubated for 2 hrs at room temperature (RT). Next, conjugate-detection solutions were added to each well, and the plates were further incubated for 2 hrs at RT. Then, substrate solution was added to each well and plates were incubated for 30 minutes. Finally, the stop solution was added to each well and the reaction was read at an optical density of 450 nm after 30 minutes using a λ correction of 540 and 570 nm. ELISA assays were capable of detecting > 10 pg/ml of each chemokine.

### RNA isolation and gene expression analysis

RNA was isolated from spleen, iliac lymph node, and urinary bladder leukocytes from CYP-induced and unaffected mice, using Tri-reagent™ (Molecular Research Center, Cincinnati, OH), according to the manufacturer's protocols. Potential genomic DNA contamination was removed from these samples by treating them with RNase-free DNase (Invitrogen, San Diego, CA) for 15 mins at 37°C. RNA was precipitated and resuspended in RNA Secure™ (Ambion, Austin, TX) and cDNA was generated by reverse transcribing approximately 1.5 μg of total RNA using Taqman™ reverse transcription reagent (Applied Biosystems, Foster City, CA), according to the manufacturer's protocols. Mouse mRNA sequences of CXCL9, CXCL10, CXCL11, CXCR3, IFN-γ, IL-12p40, TNF-α, and 18S rRNA were obtained from the NIH-National Center for Biotechnology Information (NCBI) gene bank database accession numbers M33266, NM_008599, NM_019494, AF045146, NM_008337, M86671, NM_013693, and X00686.1. These sequences were used to design primers for real-time polymerase chain reaction (RT-PCR) analysis, which generated amplicons of 100, 95, 93, 96, 98, 97, 102, and 149 base-pair fragments of CXCL9, CXCL10, CXCL11, TNF-α, IFN-γ, IL-12p40 and CXCR3 mRNAs, and 18S rRNA, respectively.

Primers were designed using the Primer 3 software program from Whitehead Institute at the Massachusetts Institute of Technology (MIT) (Boston, MA). Thermodynamic analysis of the primers was conducted using the following computer programs: Primer Premier™ (Integrated DNA Technologies, Coralville, Iowa) and MIT Primer III (Boston, MA). The resulting primer sets were compared against the entire murine genome to confirm specificity and ensure that the primers flanked mRNA splicing regions. cDNA was generated, as before, and amplified with specific cDNA primers using SYBR^® ^Green PCR master mix reagents (Applied Biosystems). Numbers of mRNA copies (> 10) relative to 18S rRNA copies were evaluated by RT-PCR analysis using the Bio-Rad Icycler and software (Hercules, CA).

### Histological evaluation

Five days after CYP (or vehicle alone) administration, mice were sacrificed and urinary bladders were fixed, sectioned at 6 μm, and stained with hematoxylin and eosin. The sections were microscopically examined at magnifications of 40× and 100× and scored for the severity of cystitis. The data presented are the mean of the number and percentage of changes ± SD of these experiments. Urinary bladders were preserved using 10% neutral formalin fixative for 24 hrs and embedded in paraffin. Fixed tissues were sectioned at 6 μm, stained with hematoxylin and eosin, and examined by light microscopy. The inflammatory state of each urinary bladder was characterized and scored as follows: having no change when compared with tissue samples from untreated mice (score = 0); having a few multi-focal mononuclear cell infiltrates (score = 1); having minimal hyperplasia with a mixture of mononuclear and multi-nucleated cells (score = 2); having major hyperplasia (score = 3) or having major hyperplasia and epithelial erosions with inflammation in the sub-mucosa (score = 4).

### Cell isolation and flow cytometry

Single-cell suspensions of spleens and iliac lymph nodes from each mouse were passed through a sterile wire screen (Sigma). Cell suspensions were washed twice in RPMI 1640. For the isolation of leukocytes from urinary bladders, this tissue was cut into small fragments and incubated for 1 hour at 37°C in RPMI 1640 containing 0.8 g/ml of collagenase II. The cell pellet was washed with medium, filtered, washed and resuspended in Hank's buffered salt solution. The cell suspension was further purified using a discontinuous Percoll (Pharmacia, Uppsala, Sweden) gradient and collected at the 40% to 75% interface. Lymphocytes were maintained in complete medium, which consisted of RPMI 1640 supplemented with 10 ml/L of nonessential amino acids (Mediatech, Washington, DC), 1 mM sodium pyruvate (Sigma), 10 mM HEPES (Mediatech), 100 U/ml penicillin, 100 μg/ml streptomycin, 40 μg/ml gentamycin (Elkins-Sinn, Inc., Cherry Hill, NJ), 50 μM mercaptoethanol (Sigma), and 10% fetal bovine serum (FBS) (Sigma). The cells were stained with phycoerythrin chychrome-5 (PE-CY-5)-conjugated anti-CD4 (H129.19), allophycocyanin (APC)-conjugated anti-LY6G (RB6-8C5), PE-conjugated anti-CD8 (LY-2 53-6.7), -CD19 (1D3), -CD11c or -DX-5 (DX5), APC-conjugated rat anti-mouse CD117, APC-conjugated anti-mouse CD34, and fluorescein isothiocynate (FITC)-conjugated anti-CD3 (145-2C11) or CD11b (M1/70) Abs (BD-PharMingen San Diego CA) for 30 mins with shaking. Lymphocytes were then washed with FACS buffer (PBS with 1% BSA) and fixed in 2% paraformaldehyde (Sigma) in PBS and analyzed by flow cytometry (Becton Dickinson, San Diego, CA).

### Statistical analysis

The traditional α-value, i.e., *p *< 0.01, was used to evaluate the statistical significance of this study. The power of these studies was carried out to determine the probability (1 – β) of detecting a significant difference (δ) between control and experimental or unaffected groups or samples. The data are expressed as the mean ± SD and compared using a two-tailed paired student's *t*-test or an unpaired Mann Whitney *U*-test. The results were analyzed using Microsoft Excel (Microsoft, Seattle, WA) for Macintosh computers. Single-factor and two-factor variance ANOVA analyses were used to evaluate groups and subgroups, respectively. Kolmogorov-Smirnov (K-S) two-sample test using CELL Quest Software (BD-PharMingen) for Macintosh computers was used to compute the statistical significance between histograms.

## Results

### Serum CXCL9, CXCL10, and CXCL11 expression during IC

**Figure 1:**
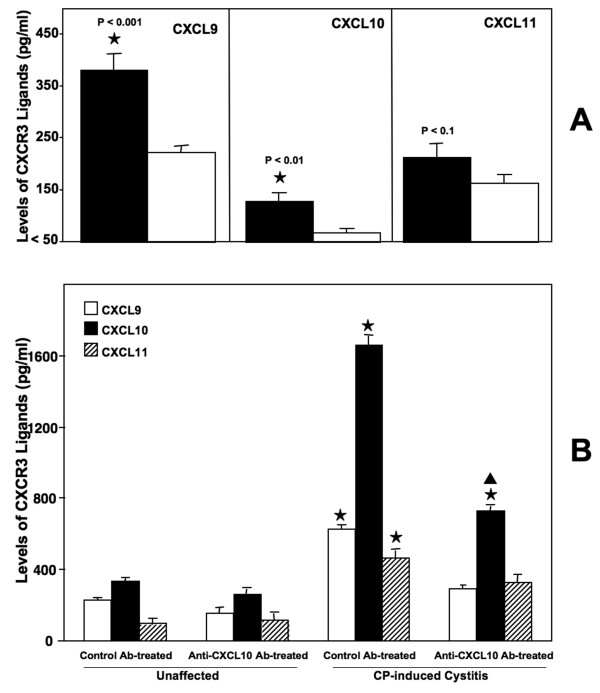
**Serum CXCL9, CXCL10 and CXCL11 concentration in the IC patients**. *Panel A*: Sera from IC patients (n = 32) and normal, healthy donors (n = 16) were isolated and evaluated for the presence of CXCR3 ligands by ELISA, capable of detecting >10 pg/ml of each CXCR3 ligand. The data presented are the mean CXCL9, CXCL10, and CXCL11 of IC patient and normal healthy donors concentrations ± SEM. Asterisks (⋆) indicate statistically significant differences, i.e., *p *< 0.01, between the healthy donors and IC patients. *Panel B*: Control or anti-CXCL10 Ab solutions were administered 2 days prior to CYP challenge and every 2 days thereafter. Five days after CYP administration, the serum levels of CXCL9, CXCL10, and CXCL11 were determined by ELISA. The data presented are the mean concentrations ± SEM in each group. Asterisks (⋆) indicate statistically significant (*p *< 0.01) differences between unaffected and CYP-induced groups. Triangles (▲) indicate statistically significant (*p *< 0.01) differences between control Ab- and anti-CXCL10 Ab-treated groups administered CYP.

The serum levels of CXCL9 and CXCL10 in IC patients were significantly higher than levels in unaffected healthy donors. In particular, the difference in serum levels between IC patients and healthy donors were greatest for CXCL9 (*p *< 0.001), followed by CXCL10 (*p *< 0.01) and CXCL11 (*p *> 0.1) (Figure 1A). These CXCR3 ligand levels also correlated (although not statistically significant) with disease severity as manifested by pathological reports for each individual patient (data not shown). These patients showed multiple pathological features of tissue damage that frequently included urothelium denudation, mucosal edema, and/or leukocyte infiltration.

### Serum CXCL9, CXCL10, and CXCL11 levels in mice after CYP induction

CYP-induced cystitis in mice led to substantial increases in serum levels of CXCL10 >> CXCL9 when compared with the levels in unaffected controls (Figure 1B). In confirmation with serum CXCR3 ligand levels in IC patients, murine CXCL11 levels did not significantly change in groups induced with CYP. In summary, mice with CYP-induced cystitis expressed higher serum CXCL10 > CXCL9 than unaffected controls, while IC patients displayed higher CXCL9 > CXCL10 serum levels than unaffected individuals.

### CXCL10 blockade effects on cystitis severity

**Figure 2:**
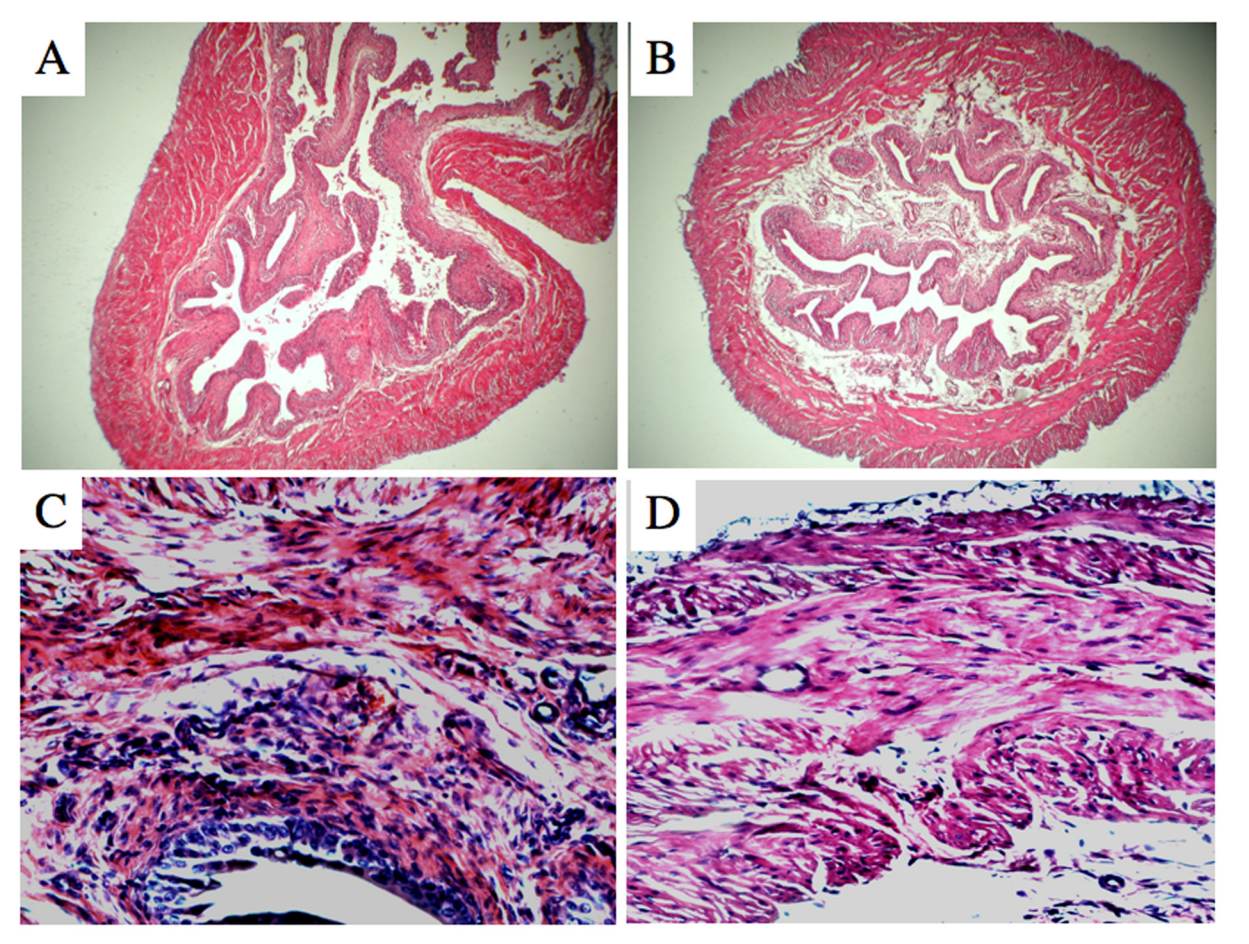
**Histological changes after CYP-induced cystitis**. Control or anti-mouse CXCL10 Ab solutions were administered 2 days prior to CYP treatment and every 2 days thereafter. Five days after CYP administration, the urinary bladders of the mice were fixed, sectioned at 6 μm, and stained with hematoxylin and eosin. The sections were examined microscopically at magnification views of 10× and 100×. *Panels A *and *C *show the magnified sections from control Ab-treated mice while *Panels B *and *D *display similar sections from anti-CXCL10 Ab-treated mice given CYP to illustrate inflamed bladders and characterized differences in mucosal wall thickness, enlargement of mucosal layer, leukocyte infiltration, and glandular elongation.

Due to the high levels of CXCL10 observed during CYP-induced cystitis, we next treated mice with control or anti-CXCL10 Abs to determine if inhibition of this chemokine would modulate disease. Unaffected mice treated with either control or anti-CXCL10 Abs showed no significant change in serum CXCR3 ligand levels. However, increases in serum CXCL10 (and CXCL9) concentrations were significantly reduced following CXCL10 blockade in mice that received CYP. Despite the reduction of serum CXCL9 and CXCL10 levels following anti-CXCL10 Ab treatment(s) of mice given CYP, it remained uncertain whether CXCL10 blockade would improve the pathology of cystitis. Control Ab-treated mice given CYP showed pathological signs of cystitis (i.e., urinary bladder inflammation, discontinuous uroepitheium). Similarly, affected mice treated with anti-CXCL10 Ab displayed a reduction in cystitis, as noted by a decrease in urinary bladder leukocyte infiltrates (Figure 2). Histological differences between control Ab- and anti-CXCL10 Ab-treated mice with CYP-induced cystitis were scored and considered significant when *p *< 0.01. In short, CXCL10 blockade significantly reduced CYP-induced cystitis (Table 1).

**Table 1:** Histological evaluation of CYP-induced cystitis

Treatment/Group	Number of Mice	Cystitis Disease Score (0 – 4)
Control Ab-treated/Naïve	15	0
Anti-CXCL10 Ab-treated/Naïve	15	0
Control Ab treated/CYP-induced	15	3.7 ± 0.4*
Anti-CXCL10 Ab-treated/CYP-induced cystitis	15	1.6 ± 0.6*^,§^

The change in urinary bladder inflammation (i.e., cystitis disease score) was evaluated by histopathology. Control or anti-mouse CXCL10 Ab solutions were administered beginning at 2 days prior to CYP (or vehicle alone) treatment and every 2 days thereafter. Five days after CYP (or vehicle alone) administration, mice were sacrificed and urinary bladders were fixed, sectioned at 6 μm, and stained with hematoxylin and eosin. The sections were examined microscopically (at 40× magnification) and scored for the severity of cystitis. The studies were repeated three times, and the data presented are the mean of the cystitis disease score ± standard deviation. Differences between CYP-induced groups and unaffected groups were considered significant when p < 0.01 (*). Differences between control and anti-CXCL10 Ab-treated mice with CYP-induced cystitis were considered significant when p < 0.01 (§).

### CXCL9, CXCL10, CXCL11, and CXCR3 mRNA expression

**Figure 3:**
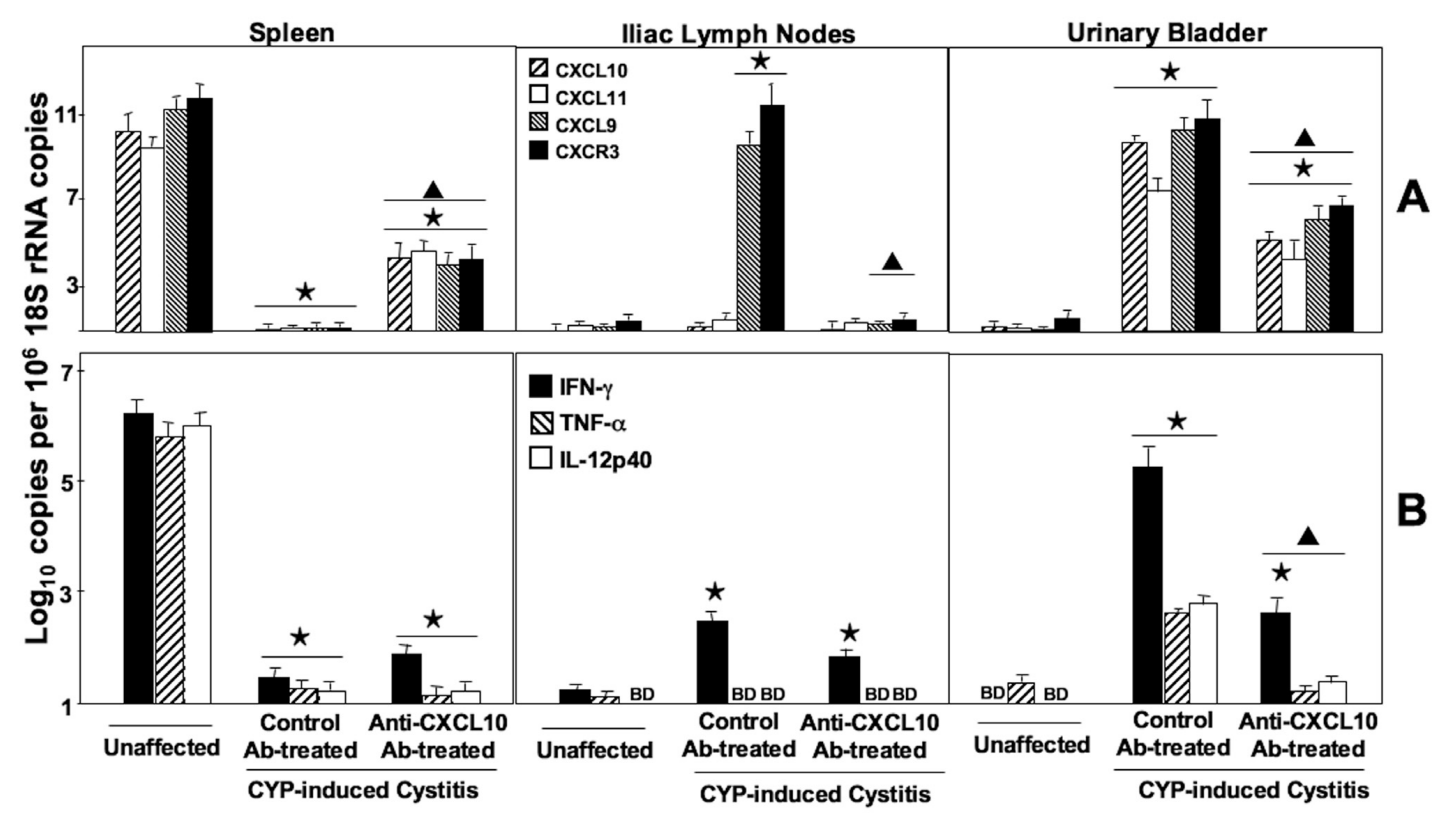
**CXCR3, CXCL9, CXCL10, and CXCL11 mRNAs expression by CYP-treated mice**. Control or anti-mouse CXCL10 Ab solutions were administered 2 days prior to CYP treatment and every 2 days thereafter. Five days after CYP administration, total RNA was isolated from the spleen, iliac lymph nodes, or urinary bladder of the mice. *Panel A*: RT-PCR analysis of CXCR3, CXCL9, CXCL10, or CXCL11 mRNA expression was performed. *Panel B*: RT- PCR analysis of IFN-γ, IL-12 p40, or TNF-α mRNA expression was performed. Log_10 _copies of transcripts ± SEM are expressed relative to actual copies of 18S rRNA. Asterisks (⋆) indicate statistically significant (*p *< 0.01) differences between unaffected and CYP-induced groups. Triangles (▲) indicate statistically significant (*p *< 0.01) differences between control Ab- and anti-CXCL10 Ab-treated groups administered CYP.

CYP-induced cystitis in mice led to substantial increases in the expression of CXCL10, CXCL11, and CXCR3 mRNA by urinary bladder leukocytes as well as modest increases in the expression of CXCL9 and CXCR3 transcripts by iliac lymph node lymphocytes than compared to normal, untreated mice (Figure 3A). In contrast, the expression of these IFN-γ – and nuclear factor kappa B (NFκB)-inducible chemokines and CXCR3 mRNAs were significantly diminished in splenocytes from CYP-treated mice than compared to similar cells from control mice. Anti-CXCL10 Ab treatment significantly decreased the expression of CXCL9 and CXCR3 mRNAs by iliac lymph node leukocytes and reduced the production of CXCL9, CXCL10, CXCL11, and CXCR3 mRNAs by urinary bladder leukocytes. However, this treatment increased the expression of these transcripts by splenocytes from CYP-induced mice than compared to controls.

### IFN-γ, IL-12 p40, and TNF-α mRNA expression

To investigate local and peripheral changes in Th1 and inflammatory cytokine expression during CYP-induced cystitis, the levels of IFN-γ, IL-12p40, and TNF-α mRNAs expressed by leukocytes isolated from the spleen, iliac lymph nodes and urinary bladder were measured by quantitative RT-PCR analysis. CYP-induced mice receiving control Ab exhibited substantial decreases in the expression of IFN-γ, IL-12p40, and TNF-α mRNAs by splenocytes; however, this treatment significantly increased the expression of cytokines by urinary bladder leukocytes than compared to unaffected mice (Figure 3B). Mice with CYP-induced cystitis exhibited increased IFN-γ mRNA expression by iliac lymph node lymphocytes compared to similar cells from unaffected mice. However, the expression of IFN-γ, IL-12p40, and TNF-α mRNAs by urinary bladder lymphocytes from mice with cystitis were significantly decreased following anti-CXCL10 Ab treatment than compared to similar cells from CYP-induced mice treated with control Ab.

### Changes in leukocyte subpopulations during cystitis

**Figure 4:**
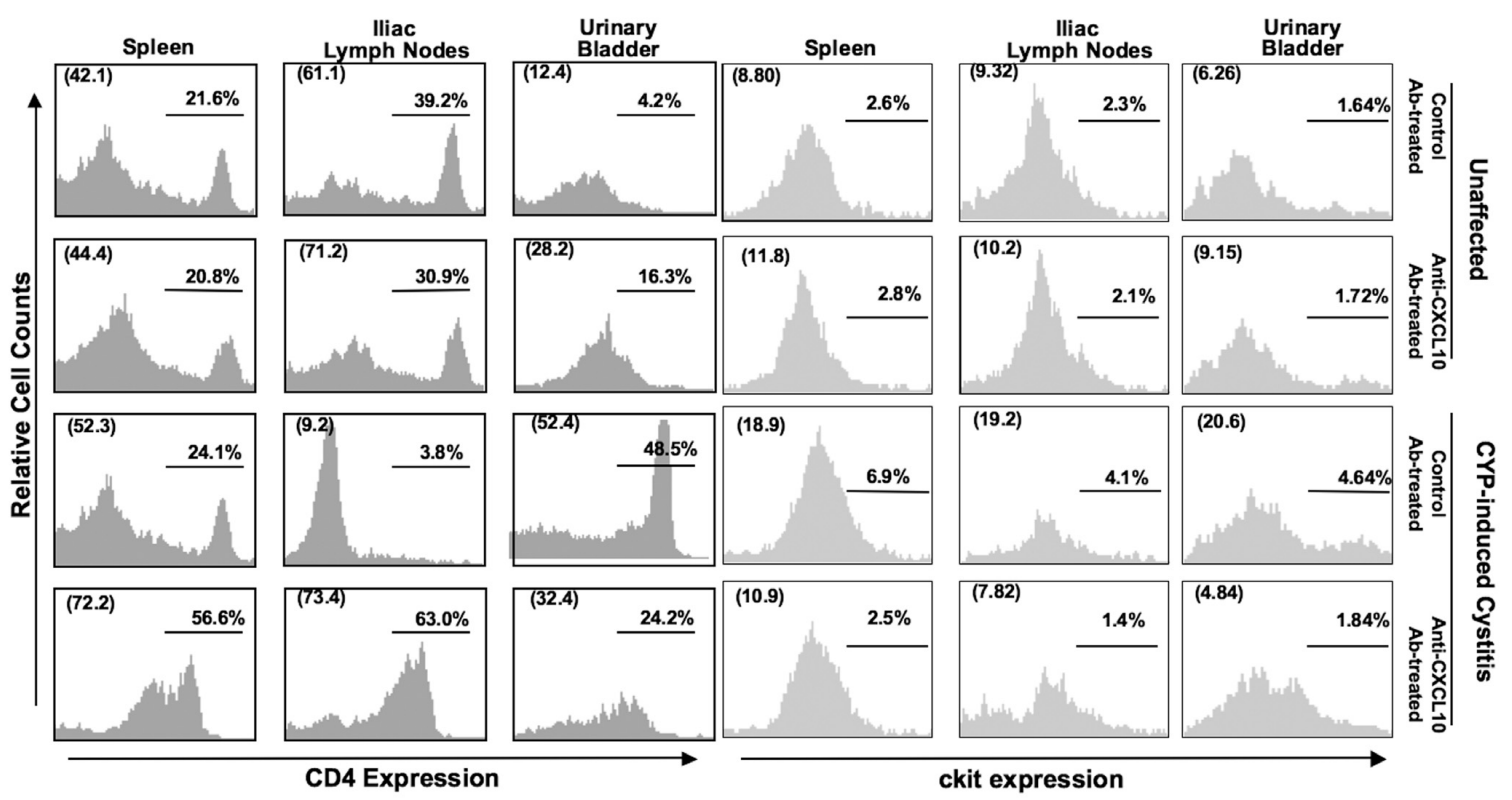
**Change in CD4^+ ^T and mast cells during acute cystitis**. Control or anti-mouse CXCL10 Ab solutions were administered 2 days prior to CYP treatment and every 2 days thereafter. Five days after CYP administration, lymphocytes from the spleen, iliac lymph nodes, and urinary bladder were isolated and stained for flow cytometry analysis. Representative histograms are shown with the mean percentage (and total number) of spleen, iliac lymph node, and urinary bladder CD4^+ ^T cells (*Panel A*), CD34^+ ^cKit^+^cell (*Panel B*).

In general, the distribution of CD3^+^CD8^+^, DX-5^+^, and CD11b^+ ^lymphocytes changed little after CYP-induced cystitis. However, significant changes of CD4^+ ^T cells were noted in urinary bladders, iliac lymph nodes, and spleens relative to unaffected mice (control or anti-CXCL10 Ab-treated) (Figure 4 and Table 2). After the induction of cystitis with CYP, the percentage of CD4^+ ^T cells decreased from 21.6% to 2.4% of the total leukocyte population in the spleen. Following anti-CXCL10 Ab treatment, the percentage of CD4^+ ^T cells (i.e., 56.6%) in total splenocytes was significantly higher. CYP treatment also lead to a decrease in the percentage of CD4^+ ^T cells from 39.3% to 3.9% in control Ab-treated mice; following anti-CXCL10 Ab treatment of CYP-challenged mice, the percentage of CD4^+ ^T cells in iliac lymph nodes increased to 63% of total lymphocytes isolated from this lymphoid tissue. CD4^+ ^T cells comprised 4.2% of the lymphocytes taken from the urinary bladder of unaffected mice. However, CYP treatment significantly increased the percentage of urinary bladder CD4^+ ^T cells to 50.5%, but CXCL10 blockade of CYP-treated mice decreased this percentage to 34.2%.

**Table 2:** Changes in the number and percentage of CD4^+ ^T cell lymphocytes.

Total CD4^+ ^T cell	Group	Spleen	Iliac lymph nodes	Urinary bladder
Counts	Control Ab-treated	24.2 × 10^6 ^± 3.9	11.0 × 10^5 ^± 0.9	1.49 × 10^4 ^± 0.04
	Anti-CXCL10 Ab-treated	22.0 × 10^6 ^± 3.1	16.4 × 10^5 ^± 1.7	2.32 × 10^4 ^± 0.2
	CYP- and control Ab-treated	0.6 × 10^6 ^± 0.06*	0.1 × 10^5 ^± 0.02	36.8 × 10^4 ^± 4.6*
	CYP- and anti-CXCL10 Ab-treated	5.2 × 10^6 ^± 0.6	1.1 × 10^5 ^± 0.1*	22.9 × 10^4 ^± 3.3*

Percentage	Control Ab-treated	21.6 ± 1.3	39.3 ± 4.4	4.24 ± 0.23
	Anti-CXCL10 Ab-treated	20.8 ± 3.8	30.9 ± 3.2	16.3 ± 2.1*
	CYP- and control Ab-treated	2.4 ± 1.9	3.9 ± 0.19	50.5 ± 7.1
	CYP- and anti-CXCL10 Ab-treated	56.6 ± 6.5*	63.0 ± 7.2*	34.2 ± 3.7*

Control or anti-mouse CXCL10 Abs solutions were administered beginning 2 days prior to CYP treatment and every 2 days thereafter. Five days after CYP administration, lymphocytes from the spleen, iliac lymph nodes, and urinary bladder were isolated, stained for CD3 and CD4 expression, and analyzed by flow cytometry. The studies were repeated three times, and the data presented are the mean of the number or percent change ± SEM (n = 10). Differences between experimental groups were considered significant when *p *< 0.01 (*).

Analysis of the change in the total number of CD4^+^ T cells in the spleen, iliac lymph nodes, and urinary bladder told a similar story. The total number of splenic CD4^+ ^T cells from CYP-induced mice decreased following CXCL10 blockade. Indeed, the effects of anti-CXCL10 Ab treatment were most evident when comparing the change in the number of CD4^+ ^T cells from control Ab *versus *anti-CXCL10 Ab treatment in the iliac lymph nodes (0.1 × 10^5 ^*versus *1.1 × 10^5^) and spleen (0.6 × 10^6 ^*versus *5.2 × 10^5^). Considered together, these findings show CYP-induced cystitis is associated with considerable reduction in the percentage of T helper lymphocytes in the spleen and iliac lymph nodes, but an increase in both the percentage and the number of CD4^+ ^T cells in the urinary bladder. Most importantly, our data show CXCL10 blockade partially attenuates these changes.

### Mast cell changes during cystitis

Mast cells have been suggested to play a role in a subset of IC cases. So, we next examined the changes in mast cells during CYP-induced cystitis. Normal mice treated with control or anti-CXCL10 Abs did not show significant changes in the percentage of mast cells taken from the spleen, urinary bladder, and iliac lymph nodes (Figure 4). Mast cells were significantly increased in the spleen, iliac lymph nodes, and urinary bladder after CYP induction than compared to control animals. However, these populations were significantly reduced after anti-CXCL10 Ab treatment.

**Figure 5:**
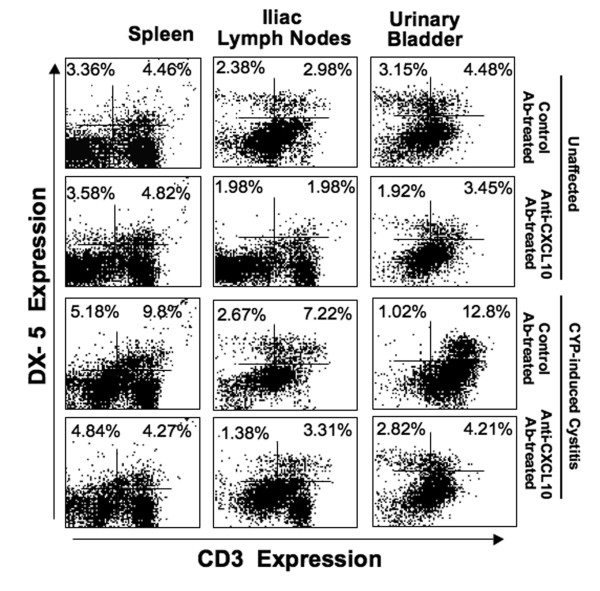
**Change in NK and NKT cells during acute cystitis**. Control or anti-mouse CXCL10 Ab solutions were administered 2 days prior to CYP treatment and every 2 days thereafter. Five days after CYP administration, lymphocytes from the spleen, iliac lymph nodes, and urinary bladder were isolated, stained for DX5 and CD3 expression and analyzed by flow cytometry. Density blots show cell distribution and indicate the percentage of DX5^+ ^CD3^- ^(NK) and DX5^+ ^CD3^+ ^(NKT) cells, respectively, present in leukocytes isolated from the spleen, iliac lymph node, and urinary bladder.

While NK cell subsets have not been attributed to either CYP-induced cystitis or IC, analysis of these leukocytes revealed the number and percentage of NK and NKT cells were elevated during CYP-induced cystitis than compared to normal control mice (Figure 5). CXCL10 blockade reduced the number of splenic, iliac lymph node, and urinary bladder NKT cells after CYP induction as compared to CYP-induced and control Ab treated mice. Splenic NK cells were unchanged during cystitis, but CXCL10 blockade dramatically reduced the number of splenic and iliac lymph node NK cells that were increased during this disease. Together, these results indicate that CXCL10 blockade modulates the number of systemic and mucosal NK cell subsets that are altered during CYP-induced cystitis.

## Discussion

At least two variants of IC have been previously described [24-26]: classical IC and non-ulcerative IC. Patients with non-ulcerative IC have relatively unaltered mucosa with sparse inflammation, multiple small mucosal ruptures, and suburothelial hemorrhages. In general, non-ulcerative IC patients show a disruption of the urothelium, vascular damage and mucosal mastocyte infiltration [27]. The four major pathology metrics of classical IC severity are mast cell count, granulation tissue, vascular density in the lamina propria and complete loss of urothelium [27-29]. However, the contribution of T helper cells as well as cytokines and chemokines in IC development remains uncertain.

While there are no mouse models that precisely mimic IC, the CYP-induced cystitis mouse model used in this study share some features of the human disease including: urinary bladder inflammation, mastocyte infiltration, and urothelium disruption. Perhaps in association with the differences between the various forms of IC and murine CYP-induced cystitis, CXCL10 (followed by CXCL9) was higher in mice with CYP-induced cystitis, but CXCL9 (followed by CXCL10) was found to be higher in IC patients. There is no clear explanation for these differences, especially considering the high identity and homology between human and mouse mature CXCL9 (75% and 89%, respectively) as well as CXCL10 (71% and 82%, respectively) proteins. However, the human *cxcl9 *promoter has six IFN-γ response elements (γ-IRE), while the mouse *cxcl9 *promoter has three. Interestingly, the mouse cxcl10 promoter has seven γ-IRE, but human CXCL10 only has three. Taken together, the higher expression of CXCL9 in man and CXCL10 in mouse, during clinical and experimental disease, respectively, could be explained by these promoter differences or the imperfect correlation between clinical disease and the experimental mouse model used in this study. Further studies will be required to precisely dissect the molecular mechanisms behind these differences.

CD4^+ ^T cell infiltration and expression of CXCL10 is seen in many mucosal inflammatory diseases [30-43]. In the current study, we show for the first time that serum levels of CXCR3 ligands are elevated in IC patients compared with normal, healthy donors. We also demonstrate for the first time that anti-CXCL10 Ab treatment prevents the development and onset of CYP-induced cystitis. The ability of CXCL10 blockade to modulate local chemokines as well as Th1 cytokine mRNA expression and to facilitate the decline of serum levels of CXCL9 and CXCL10 is an indication of reduced local as well as systemic inflammation. This study supports the previous finding that there are elevated numbers of mucosal T cells during IC pathogenesis [44].

Our results show that CXCL10 blockade does not affect the percentage of CD4^+ ^T cells in the spleen of naïve mice, but inhibition of CXCL10 in CYP-treated mice with cystitis lead to an increase in the percentage of T helper splenocytes. We believe these findings are a result of numerous interactions including the ability of CYP to selectively inhibit the proliferation of T regulatory (Treg) cells without affecting Th1 cells that are typically CXCR3^+^. The presence of anti-CXCL10 Ab might hinder the movement of these CXCR3^+ ^T cells that would otherwise migrate to the urinary bladder and/or iliac lymph nodes to exacerbate CYP-induced cystitis. To explain, CYP is an "alkylating agent" that binds DNA and disrupts cell division. Rapidly dividing cells are especially sensitive to CYP. The mechanism by which CYP modulates inflammation might be due to the cytotoxic effect of CYP on proliferating CD25^+ ^T cells (e.g., Treg cells). Indeed, CYP has been shown to selectively target Treg cells [45] and lead to their reduction in number [46]; CD4^+ ^CD25^+ ^T cells isolated from CYP-treated mice display reduced suppressive activity *in vitro *and increased expression of apoptotic markers. A large body of evidence describes the mechanisms of immune response augmentation by CYP, which include the homing and homeostatic proliferation by the creation of space, the skewing of Th2/Th1 responses due to the cytokine storm during the recovery phase, and the removal or inhibition of Treg cells [47,48]. In this regard, because of the ability of CYP to dysregulate the immune system, it has been used to induce as well as treat immune disorders. For example, CYP has been shown to increase the number of Th2 cells in a model of multiple sclerosis [49]. In contrast, CYP is used to induce type1 diabetes via the promotion of autoimmune Th1 cells and presumably suppression of Treg cells [50,51].

It is important to mention that acrolein, the highly reactive aldehyde derived from the metabolism of CYP, is responsible for the cystitis associated with CYP administration [13]. Acrolein treatment causes loss of urothelium, which is followed by leukocyte infiltration to the bladder. Additional studies will be required to determine the exact mechanism(s) leading to CYP- and/or acrolein-induced cystitis in mice; however, the current study provides some explanations for some insight into the molecular and cellular mechanisms responsible for these changes and the role of CXCL10 in CYP-induced cystitis. It has been reported that CXCL10 triggers lymphocyte adhesion to immobilized integrin ligands [52] and enhances colitis through a massive infiltration of CD4^+ ^T cells, which produce Th1 cytokines [53,54]. Environmental (e.g., acrolein) or pathogenic (e.g., *Escherichia coli*) insults induce epithelial cells to increase their expression of CXCL10 [55], suggesting the mucosa participates in modulating T cell-mediated inflammation via CXCR3-CXCR3 ligand interactions.

The role of CXCL10 has been contributed to be an important hallmark of in the of inflammatory bowel disease (IBD) pathophysiology [39-42]. Blockade of CXCL10 ameliorated murine AIDS and acute colitis by inhibiting cell trafficking as well as inhibiting cell proliferation while supporting crypt epithelial cell survival [56,57]. Indeed, CXCL10 inhibition might prove useful in other diseases. For example, *lichen sclerosus et atrophicus *or white-spot disease is a chronic inflammatory skin disease where CXCR3^+ ^cytotoxic T cells are involved in its pathogenesis [58]. These cells also contain CXCL10 in their granules to perpetuate this disease once released. Similar observations have been made in oral *lichen planus *[59].

TNF-α is a potent inducer of CXCL10 expression by endothelial cells [60]; this inflammatory cytokine was negatively modulated by CXCL10 blockade during CYP-induced cystitis. The significant role of TNF-α in CYP-induced cystitis has been reported in the past [61] and high levels of TNF-α have been observed in the urine of IC patients [62,63]. The described study shows that anti-CXCL10 Ab therapy reduces TNF-α expression that correlates with decreased cystitis severity. Thus, the decline in inflammatory cytokines levels might be a reflection of reduced inflammatory state and Th1 cell-mediated responses afforded by CXCL10 blockade.

Mast cells are normally distributed throughout the mucosal surface and connective tissues and have the capacity to release or synthesize numerous inflammatory mediators including histamine, TNF-α, and prostaglandins [64]. Mast cells have been implicated in a subset of IC cases [65] and involved in ulcerative and non-ulcerative IC [66,67]. In the present study, we noticed significant increases in mast cell numbers in the spleen, iliac lymph nodes, and urinary bladder after CYP induction and these increases were abrogated by anti-CXCL10 Ab treatment. Taken together, the present study presents a scenario where CYP induction leads to increased numbers of both systemic and mucosal mast cells, partially through CXCL10-CXCR3 interactions.

While NK cell subsets have not been demonstrated to play a profound role in IC or in CYP-induced cystitis, this study shows that NK and NKT cells were associated with cystitis in mice following CYP challenge, which was partially resolved by CXCL10 blockade. NK and NKT cells express CXCR3 and have been shown to be involved in the differentiation of naïve CD4^+ ^T cells to Th1 cells [68]. Importantly, the regulatory role of these cells may be crucial for controlling the extent of Th1 cells activation and progression during CYP-induced cystitis. Additional studies will be required to dissect the mechanisms of NK cell-mediated cystitis.

The ratio of inflammatory Th1 to Treg cells increases during IC [69]. Importantly, CXCR3-expressing T cells have been shown to predominantly produce Th1 cytokines and selectively mobilize Th1 and inflammatory lymphocytes [70]. In the present study, we observed a significant increase in the number of CD4^+ ^T cells at the site of urinary bladder inflammation in CYP-induced, control Ab-treated mice than compared to similar mice treated with anti-CXCL10 Ab. Furthermore, we observed a significant decline in IFN-γ and IL-12p40 mRNA expression by urinary bladder lymphocytes from mice challenged with CYP and treated with anti-CXCL10 Ab, than compared to control Ab-treated mice. CXCL10 blockade reduced the number of CD4^+ ^T cells at this effector site, most likely by inhibiting chemotaxis and activation via CXCR3 interactions. When considered together, the results of the present study suggest that CYP-induced cystitis is partially Th1-cell- and CXCR3 ligand-mediated.

## Conclusion

In this mouse model, IFN-γ transcripts were not significantly expressed by the iliac lymphocytes during CYP-induced cystitis but were expressed by leukocytes from the spleen and urinary bladder. The expression of CXCL9, CXCL10, and CXCL11 can be induced by IFN-γ [71]. Hence, the current study suggests CXCL10 is partially responsible for changes in the distribution of CXCR3^+ ^Th1 cells, mast cells, and NKT cells from the spleen and iliac lymph nodes to the urinary bladder after CYP treatment, which corresponded with cystitis. Flow cytometry analysis showed the percentage of CD4^+ ^T cells, and NK cells was dramatically lower in the iliac lymph nodes of CYP-treated mice than in the iliac lymph nodes of CYP-induced and anti-CXCL10 Ab-treated mice. In contrast, the number of CD4^+ ^T cells, mast cells, and NKT cells were dramatically changed in the urinary bladders of CYP-treated mice than compared to the number of similar cells isolated from urinary bladders. The present study suggests a role for CXCL10 as a mediator of leukocyte infiltration to the urinary bladder (effector site) from the iliac and peripheral lymphoid tissues (inductive sites) during cystitis. While anti-CXCL10 Ab therapy has not been used to treat IC, this approach (i.e., MDX-1100) has been proven to be safe and well tolerated in the treatment of IBD, clearing Phase I testing with no serious side effects [72,73]. Considered together, the CXCR3-CXCL10 axis may represent a new target for the treatment of cystitis.

## Abbreviations

Ab: antibody; BrdU: 5-Bromo-2'-deoxy-uridine; CD: Crohn's disease; CXCL9, MIG: monokine-induced by IFN-γ; CX3CL1: fractalkine; CXCL10, IP-10: IFN-γ-inducible protein 10; CXCL11, I-TAC: IFN-γ-inducible T cell-α chemoattractant; CYP: cyclophosphamide; DCs: dendritic cells; ELISA: enzyme-linked immuno-sorbent assay; IC: interstitial cystitis; IFN: interferon; IL: interleukin; NK: natural killer; NKT: natural killer T (cell): SAA: serum amyloid A; STAT: signal transducers and activators of transcription; Th: T helper; TNF: tumor necrosis factor; Treg: T regulatory.

## Competing interests

The authors declare that they have no competing interests.

## Authors' contributions

US and SS carried-out all animal studies, quantified serum CXCR3 ligands, performed flow cytometry acquisition and analyzed data with the assistance of SS. DT and KN coordinated and performed the CXCR3 ligand and SAA serum ELISA of IC patients. JL conceived the study, participated in its design with all authors, coordinated and helped to draft the manuscript with the assistance of all authors. All authors read and approved the final manuscript.
